# SARS Control and Psychological Effects of Quarantine, Toronto, Canada

**DOI:** 10.3201/eid1007.030703

**Published:** 2004-07

**Authors:** Laura Hawryluck, Wayne L. Gold, Susan Robinson, Stephen Pogorski, Sandro Galea, Rima Styra

**Affiliations:** * University of Toronto, Toronto, Ontario, Canada;; † ASK Information Technologies, Toronto, Ontario, Canada;; ‡ New York Academy of Medicine, New York, New York, USA

**Keywords:** SARS, quarantine, post–traumatic stress disorder, depression

## Abstract

As a transmissible infectious disease, severe acute respiratory syndrome (SARS) was successfully contained globally by instituting widespread quarantine measures. Although these measures were successful in terminating the outbreak in all areas of the world, the adverse effects of quarantine have not previously been determined in a systematic manner. In this hypothesis-generating study supported by a convenience sample drawn in close temporal proximity to the period of quarantine, we examined the psychological effects of quarantine on persons in Toronto, Canada. The 129 quarantined persons who responded to a Web-based survey exhibited a high prevalence of psychological distress. Symptoms of posttraumatic stress disorder (PTSD) and depression were observed in 28.9% and 31.2% of respondents, respectively. Longer durations of quarantine were associated with an increased prevalence of PTSD symptoms. Acquaintance with or direct exposure to someone with a diagnosis of SARS was also associated with PTSD and depressive symptoms.

## Abstract 2

Explores effects of quarantine on those quarantined for SARS, Toronto, Canada

Severe acute respiratory syndrome (SARS) was contained globally by widespread quarantine measures, measures that had not been invoked to contain an infectious disease in North America for >50 years (*1**–**6*). Although quarantine has periodically been used for centuries to contain and control the spread of infectious diseases such as cholera and the plague with some success (*1**–**4**,**6**–**8*), the history of invoking quarantine measures is tarnished by threats, generalized fear, lack of understanding, discrimination, economic hardships, and rebellion (*1**,**3**,**4**,**6**–**8*).

Quarantine separates persons who have been potentially exposed to an infectious agent (and thus at risk for disease) from the general community. For the greater public good, quarantine may create heavy psychological, emotional, and financial problems for some persons. To be effective, quarantine demands not only that at-risk persons be isolated but also that they follow appropriate infection control measures within their place of quarantine. Reporting on SARS quarantine has focused on ways in which quarantine was implemented and compliance was achieved (*1**–**4**,**6**–**8*). Adverse effects on quarantined persons and the ways in which those quarantined can best be supported have not been evaluated. Moreover, little is known about adherence to infection-control measures by persons in quarantine.

Knowledge and understanding of the experiences of quarantined persons are critical to maximize infectious disease containment and minimize the negative effects on those quarantined, their families, and social networks. The objectives of our study were to assess the level of knowledge about quarantine and infection control measures of persons who were placed in quarantine, to explore ways by which these persons received information to evaluate the level of adherence to public health recommendations, and to understand the psychological effect on quarantined persons during the recent SARS outbreaks in Toronto, Canada.

## Methods

### Description of Quarantine in Toronto

During the first and second SARS outbreaks in Toronto, >15,000 persons with an epidemiologic exposure to SARS were instructed to remain in voluntary quarantine (Health Canada, unpub. data). Data on the demographics of the quarantined population were collected, but have not yet been analyzed (B. Henry, Toronto Public Health, pers. comm.). Quarantined persons were instructed not to leave their homes or have visitors. They were told to wash their hands frequently, to wear masks when in the same room as other household members, not to share personal items (e.g., towels, drinking cups, or cutlery), and to sleep in separate rooms. In addition, they were instructed to measure their temperature twice daily. If any symptoms of SARS developed, they were to call Toronto Public Health or Telehealth Ontario for instructions (*5*).

### Study Population

All persons who were placed in quarantine during the SARS outbreaks in Toronto (at least 15,000 persons) were eligible for participation in this study. The survey was announced through media releases, including locally televised interviews with the principal investigators. Information on the study and invitations to participate were posted in local healthcare institutions, libraries, and supermarkets. Ethics approval was obtained from the research ethics board of the University Health Network, a teaching institution affiliated with the University of Toronto.

### Survey Instrument

A Web-based survey composed of 152 multiple choice and short- answer questions was to be completed after participants ended their period of quarantine. It took approximately 20 minutes to complete. Questions explored included the following: 1) knowledge and understanding of the reasons for quarantine (*2*), knowledge of and adherence to infection control directives, and (*3*) source of this knowledge.

The psychological impact of quarantine was evaluated with validated scales, including the Impact of Event Scale—Revised (IES-R) (*9*) and the Center for Epidemiologic Studies—Depression Scale (CES-D) (*10*). The IES-R is a self-report measure designed to assess current subjective distress resulting from a traumatic life event and is composed of 22 items, each with a Likert rating scale from 0 to 4. The maximum score is 88. In a study of journalists working in war zones, the mean IES-R score of posttraumatic stress disorder (PTSD) was 20. In these persons, the presence of PTSD symptoms, as measured by this scale, was correlated with diagnostic psychiatric interviews (*11*). The CES-D is a measure of depressive symptoms composed of 20 self-report items, each with a Likert rating scale from 0 to 3. The maximum score is 60 (*10*). A score of> 16 has been shown to identify persons with depressive symptoms similar in severity to the levels observed among depressed patients (*10**,**12**,**13*). Open-ended questions provided respondents with the opportunity to relate the aspects of quarantine that were most difficult for them and allowed them to provide additional comments on their unique experiences.

### Statistical Analysis

Means were calculated to summarize continuous variables. For categorical variables, group proportions were calculated. Student *t* tests were used to examine relationships between demographic variables and the psychological outcome variables, the scores on the IES-R and CES-D. A score of >20 on the IES-R was used to estimate the prevalence of PTSD symptoms (*11*). A score of >16 on the CES-D was used to estimate the prevalence of depressive symptoms (*10**,**12**,**13*).

Analysis of variance (ANOVA), chi-square, and the Cochran-Armitage test for trend were used to examine relations between the IES-R and CES-D scores and the following independent variables: healthcare worker status, home or work quarantine, acquaintance of or direct exposure to someone with a diagnosis of SARS, combined annual household income, and the frequency with which persons placed in quarantine wore their masks. Linear regression for the trends between income categories and both PTSD and depressive symptoms was analyzed. The relationships between the IES-R and CES-D and whether persons in quarantine wore their masks all of the time versus never were examined by the Duncan-Waller K-ratio *t* tests. A p value of < 0.05 was considered to be significant for all analyses.

Qualitative data were coded and analyzed to show emerging themes. The development and confirmation of the thematic coding structure is an iterative process involving two researchers in individual, recursive reading of the textual data and group meetings to discuss and test the emerging themes. Discrepancies were resolved by consulting specific instances in the data, discussing their relationship to established themes, and reaching consensus as a group (*14*).

## Results

### Demographics and Description of Quarantined Persons

The survey was completed by 129 of more than 15,000 eligible persons who were placed in quarantine (Figure). All respondents completed the survey at the end of quarantine with a minimum time from the end of quarantine to the completion of the survey of 2 days. The median time from the end of quarantine to completion of the survey was 36.0 days (interquartile range, 10–66 days). Sixty-eight percent of respondents were healthcare workers, 64% were 26–45 years of age, 58% were married, 72% had a college level of education or higher, and 48% had a combined household income of >$75,000 (Canadian dollars [CAD]).

**Figure:**
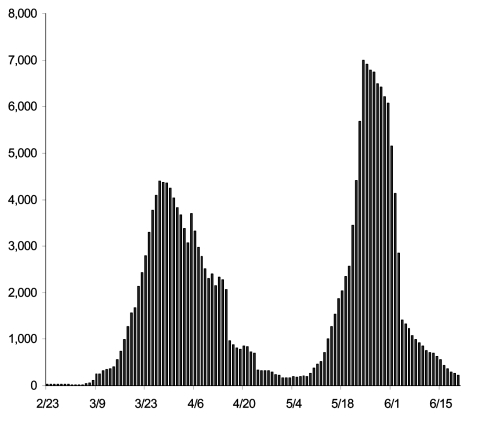
Number of persons in quarantine, Toronto, Canada, February 23–June 30, 2003. Figure courtesy of Toronto Public Health.

The 129 respondents described 143 periods of quarantine with 90% of respondents being placed into quarantine only once; 66% of respondents were on home quarantine, while 34% were on work quarantine. The median duration of quarantine was 10 days (interquartile range 8–10 days). Half of respondents knew someone who was hospitalized with SARS of whom 77% were colleagues; 10% knew someone who had died of SARS (Table 1).

**Table 1:** Characteristics of quarantined persons who responded to the survey

Characteristic	No. (%) N=129
Age (y)	
18–25	11 (8.6)
26–35	37 (28.9)
36–45	44 (34.4)
46–55	21 (16.4)
56–65	11 (8.7)
66+	4 (3.1)
Marital status	
Married or common law	87 (68.0)
Single or divorced	41 (32.0)
Education	
High school	11 (9.2)
College or university	109 (90.8)
Income (Canadian $)	
<$20,000	6 (5.8)
$20,000–$39,999	8 (8.5)
$40,000–$74,999	35 (33.0)
$75,000–$99,999	20 (18.8)
>$100,000	36 (34.0)
Healthcare worker status	
No	40 (31.8)
Yes	86 (68.3)
Type of quarantine (N = 143 episodes)	
Work	49 (34.3)
Home	94 (65.7)
Household members	
No. adults	
1	28 (21.9)
2	72 (56.4)
3	22 (17.2)
4	5 (3.9)
>5	1 (0.8)
No. children	
0	72 (55.8)
1	24 (18.6)
2	25 (19.4)
3	8 (6.2)

Persons were notified of their need to go into quarantine from the following sources: their workplace (58%), the media (27%), their healthcare provider (7%), and public health officials (9%). Most (68%) understood that they were quarantined to prevent them from transmitting infection to others; 8.5% of respondents believed they were quarantined to protect themselves from infection; 15% did not believe they should have been placed into quarantine at all; and 8.5% provided more than one of these responses.

The source of notification for quarantine influenced understanding of the reason for quarantine. Those who were notified by the media or their workplace were more likely to understand the reason for quarantine than those who were notified by their healthcare provider or public health unit (p = 0.04). Healthcare workers were also more likely to understand the reason for quarantine compared with non–healthcare workers, 76.5% versus 52.5% (p = 0.007). Combined household income and level of education did not influence understanding of the reason for quarantine.

### Information on Infection Control Measures

Persons received their information regarding infection control measures to be adhered to during their quarantine from the following sources: the media (54%), public health authorities (52%), occupational health department (33%), healthcare providers (29%), word-of-mouth (23%), hospital Web sites (21%), and other Web sites (40%).

Those who did not think they had been well-informed were angry that information on infection control measures and quarantine was inconsistent and incomplete, frustrated that employers (healthcare institutions) and public health officials were difficult to contact, disappointed that they did not receive the support they expected, and anxious about the lack of information on the modes of transmission and prognosis of SARS (Appendix).

During the outbreaks, nearly 30% of respondents thought that they had received inadequate information about SARS. With respect to information regarding home infection control measures, 20% were not told with whom they could have contact; 29% did not receive specific instructions on when to change their masks; and 40%–50% did not receive instructions on the use and disinfection of personal items, including toothbrushes and cutlery; 77% were not given instructions regarding use and disinfection of the telephone. Healthcare worker status did not influence whether respondents thought they had received adequate information regarding any of the listed home infection control measures, except regarding the frequency of mask changing: healthcare workers more frequently reported that they had received adequate information, 78.8% versus 60.5% (p = 0.03).

### Adherence to Infection Control Measures

Eighty-five percent of quarantined persons wore a mask in the presence of household members; 58% remained inside their residence for the duration of their quarantine. Thirty-three percent of those quarantined did not monitor their temperatures as recommended: 26% self-monitored their temperatures less frequently than recommended, and 7% did not measure their temperatures at all. No differences between healthcare workers and nonhealthcare workers were found with respect to adherence to recommended infection control measures.

### Psychological Impact of Quarantine

The mean IES-R score was 15.2±17.8, and the mean CES-D was 13.0±11.6. The IES-R score was >20 for 28.9%; the CES-D score was >16 in 31.2% of quarantined persons (Table 2). The mean IES-R scores were not different for persons on home or work quarantine, 14.1±18.8 versus 17.6±16.6 (p = 0.33); the mean CES-D scores were also not different between the groups, 12.0±12.0 versus 15.2±10.7 (p = 0.16).

**Table 2:** Prevalence of posttraumatic stress disorder and depressive symptoms according to patient demographics^a^

Characteristic	No. (%) N=129		
Prevalence			
CES-D			
<16	84 (68.8)		
>16	38 (31.2)		
IES-R			
<20	86 (71.1)		
>20	35 (28.9)		
Marital status	Mean	SD	p value
CES-D			
Single or divorced (n = 40)	12.9	10.7	0.85
Married (n = 79)	12.5	11.4	
IES-R			
Single or divorced (n = 39)	14.5	16.6	0.82
Married (n = 79)	13.8	14.6	
Income (Canadian $)			
CES-D			
<$40,000	18.3	15.4	0.05^b^
$40,000–$75,000	15.5	13.2	
>$75,000	10.9	9.2	
IES-R			
<$40,000	24.2	20.6	0.03^b^
$40,000–$75,000	19.9	24.4	
>$75,000	11.8	11.6	
Duration of quarantine (d)			
CES-D			
<10	11.2	10.1	0.07
>10	17.0	14.2	
IES-R			
<10	11.7	10.7	0.05
>10	23.7	27.2	

^a^CES-D, Center for Epidemiologic Studies—Depression Scale (*10*); IES-R,Impact of Event Scale—Revised (*9*). ^b^By analysis of variance.

The presence of PTSD symptoms was correlated with the presence of depressive symptoms (p < 0.0001, r = 0.78). Marital status did not offset the presence of PTSD symptoms, mean IES-R score of 14.5±16.6 for those who were unmarried versus 13.8±14.6 for those who were married (p = 0.82). Similarly, marital status did not influence the presence of depressive symptoms, with a mean CES-D score of 12.9±10.7 for those who were unmarried versus 12.5±11.4 for those who were married (p = 0.85)

A combined annual household income of CAD <$40,000 versus CAD $40,000 to CAD $75,000 versus CAD >$75,000 was associated with increased PTSD symptoms (mean IES-R score of 24.2±20.6 versus 20.0±24.4 versus 11.8±11.6, respectively) (p = 0.03 for the three-way comparison). Linear regression testing for trend over income categories was also significant (p = 0.01). A combined annual household income of CAD <$40,000 versus CAD $40,000 to CAD $75,000 versus CAD >$75,000 was also associated with increased depressive symptoms (mean CES-D score of 18.3±15.4 versus 15.5±13.2 versus 10.9±9.2, respectively) (p = 0.05 for the three-way comparison) (Table 2). Results of linear regression testing for trend over income categories were also significant (p = 0.01).

Neither age, level of education, healthcare worker status, living with other adult household members, nor having children was correlated with PTSD and depressive symptoms. The duration of quarantine was significantly related to increased PTSD symptoms, mean IES-R score of 23.7±27.2 for those in quarantine >10 days compared with 11.7±10.7 for those in quarantine <10 days (p < 0.05). Persons who were in quarantine for a longer duration showed a trend toward higher CES-D scores; however, this difference did not reach statistical significance (mean CES-D of 17.0±14.2 for those in quarantine >10 days versus 11.2±10.1 for those in quarantine <10 days [p = 0.07]). Acquaintance with or exposure to someone who was hospitalized with SARS was associated with a higher mean IES-R score, 18.6±20.2 versus 11.8±14.3 (p = 0.03) and a higher mean CES-D score, 15.5±12.1 versus 10.2±10.5 (p = 0.01). Overall, acquaintance with or exposure to someone who died of SARS was not correlated with PTSD or depressive symptoms (data not shown).

Persons were categorized as having worn their masks all of the time, including times when it was not recommended, having worn their masks according to recommendations, or not having worn their masks at all. Those who wore their masks all of the time had higher mean IES-R scores (29.7±18.6 versus 14.1±17.9 versus 12.3±15.1, p = 0.003 for the three-way comparison) and higher mean CES-D scores (25.6±12.7 versus 12.2±11.1 versus 11.5±11.6, p = 0.002 for the three-way comparison). Those who wore their masks all of the time also had higher mean IES-R scores (p = 0.03) and higher mean CES-D scores (p = 0.002) than those who never wore their masks.

All respondents described a sense of isolation. The mandated lack of social and, especially, the lack of any physical contact with family members were identified as particularly difficult. Confinement within the home or between work and home, not being able to see friends, not being able to shop for basic necessities of everyday life, and not being able to purchase thermometers and prescribed medications enhanced their feeling of distance from the outside world. Infection control measures imposed not only the physical discomfort of having to wear a mask but also significantly contributed to the sense of isolation. In some, self-monitoring of temperature provoked considerable anxiety: "taking temperatures was mentally difficult" (respondent #27) and "taking my temperature made my heart feel like it was going to pound out of my chest each time" (respondent #62).

While most quarantined persons (60%) did not believe that they would contract SARS, 59% were worried that they would infect their family members. In contrast, only 28% were concerned that a quarantined family member would infect someone else in the home. Following quarantine, 51% of respondents had experiences that made them feel that people were reacting differently to them: avoiding them, 29%; not calling them, 7%; not inviting them to events, 8%; and not inviting their families to events, 7%.

## Discussion

Persons placed in quarantine have their freedom restricted to contain transmissible diseases. This takes a considerable toll on the person. In relation to the recent global outbreak of SARS, considerable time has been spent discussing the specifics of quarantine and how to promote adherence to infection control measures. Little, if any, analysis has focused on the effect of quarantine on the well-being of the quarantined person. The objective of the study survey was to capture a range of experiences of quarantined persons to better understand their needs and concerns. This knowledge is critical if modern quarantine is to be an effective disease-containment strategy. To our knowledge, a consideration of the adverse effects of quarantine, including psychological effects, has not previously been systematically attempted.

Our results show that a substantial proportion of quarantined persons are distressed, as evidenced by the proportion that display symptoms of PTSD and depression as measured by validated scales. Although quarantined persons experienced symptoms suggestive of both PTSD and depression, the scales that were used to measure these symptoms are not sufficient to confirm these diagnoses. To confirm the diagnoses of PTSD and depression, structured diagnostic interviews are required. Because the survey was anonymous, this was not possible.

A score of >20 on the IES-R was used to estimate the prevalence of PTSD symptoms in our study population. This corresponds to the mean score measured on the IES-R in a study of journalists working in war zones that used diagnostic psychiatric interviews to confirm the presence of this disorder (*11*). Since most respondents to our survey were healthcare workers, we chose a work-related traumatic event for the comparison group. While other cutoff points may have been used to estimate the prevalence of PTSD symptoms in our population, the risk factors that we identified for increased PTSD symptoms, rather than the absolute prevalence of PTSD in our study participants, are the important findings of this study. This also applies to the risk factors that we identified for increased depressive symptoms in the respondents. Quarantined persons with risk factors for either PTSD or depressive symptoms may benefit from increased support from public health officials.

In this population, the presence of PTSD symptoms was highly correlated with the presence of depressive symptoms even though different clinical symptoms characterize the two disorders. Kessler's National Comorbidity Study indicated a 48.2% occurrence of depression in patients with PTSD (*15*).

PTSD is an anxiety disorder characterized by avoiding stimuli associated with a traumatic event, reexperiencing the trauma, and hyperarousal, such as increased vigilance (*16*). This disorder may develop after exposure to traumatic events that involve a life-threatening component, and a person's vulnerability to the development of PTSD can be increased if the trauma is perceived to be a personal assault (*17*). Increased length of time spent in quarantine was associated with increased symptoms of PTSD. This finding might suggest that quarantine itself, independent of acquaintance with or exposure to someone with SARS, may be perceived as a personalized trauma. The presence of more PTSD symptoms in persons with an acquaintance or exposure to someone with a diagnosis of SARS compared to persons who did not have this personal connection may indicate a greater perceived self-risk. The small number of respondents who were acquainted with or exposed to someone who died of SARS may explain the lack of correlation between this group and greater PTSD and depressive symptoms (44 persons died of SARS in the greater Toronto area).

This study also notes the trend toward increasing symptoms of both PTSD and depression as the combined annual income of the respondent household fell from CAD >$75,000 to CAD <$40,000. Quarantined persons with a lower combined annual household income may require additional levels of support. Since the survey was Web-based and required that respondents have access to a computer, the survey was likely answered by a more affluent and educated subgroup of persons. Since respondents with a lower combined annual household income experienced increased symptoms of PTSD and depression, and since those with lower combined annual household incomes were not as likely to have access to a computer, the results of this survey may underestimate the prevalence of psychological distress in the overall group of quarantined persons. Overall, most respondents did not report financial hardship as a result of quarantine. This finding is likely explained by the fact that >50% of the respondents reported a combined annual household income of CAD >$75,000.

As many as 50% of respondents felt that they had not received adequate information regarding at least one aspect of home infection control, and not all of the respondents adhered to recommendations. Why some infection control measures were adhered to while others were not is unclear. A combination of lack of knowledge, an incomplete understanding of the rationale for these measures, and a lack of reinforcement from an overwhelmed public health system were likely contributors to this problem. Of particular interest, strictly adhering to infection control measures, including wearing masks more frequently than recommended, was associated with increased levels of distress. Whether persons with higher baseline levels of distress were more likely to strictly adhere to infection-control measures or whether adherence to recommended infection-control strategies resulted in developing higher levels of distress cannot be clarified without interviewing the respondents. Regardless of the cause, this distress may have been lessened with enhanced education and continued reinforcement of the rationale for these measures and outreach efforts to optimize coping with the stressful event.

This study has several limitations. The actual number of respondents is low compared to the total number of persons who were placed into quarantine and therefore may not be representative of the entire group of quarantined persons. However, lack of funding, confidentiality of public health records, and an overloaded public health response system limited sampling in this study. Furthermore, a self-selection effect may have occurred with those persons who were experiencing the greatest or least levels of distress responding to the survey. In addition, respondents required access to a computer to respond, which suggests that they may be more educated and have higher socioeconomic status than the overall group who were quarantined. They also had to be English speaking. Recognizing these limitations, however, an anonymous Web-based method was chosen because concerns about persons' confidentiality precluded us from access to their public health records.

A Web-based format was chosen over random-digit dialing for both cost considerations and time constraints. The project was initiated and completed without a funding source soon after the outbreak period at a time when concerns about SARS were still a part of daily life in Toronto. Obtaining as much information about the adverse effects of quarantine as close to the event as possible was important because a study conducted several months later would have been subject to the limitations of substantial recall bias. If this study were to be repeated, a study design ensuring a more representative selection of the population that used a combination of quantitative and qualitative methods, including structured diagnostic interviews, would be recommended to overcome these concerns. In the event of future outbreaks, a matched control group of persons who were not quarantined should be considered because it would allow an assessment of the distress experienced by the community at large.

Finally, we determined only the prevalence of symptoms of PTSD and depression in our study population because these were the predominant psychological distresses that were observed to be emerging in our SARS patient population (W.L.G., pers. comm.). We also focused on symptoms of PTSD and depression because we believed that they would be the most likely to cause illness and interfere with long-term functioning. Future studies should assess persons for other psychological responses, including fear, anger, guilt, and stigmatization. A standardized survey instrument that considers the full spectrum of psychological responses to quarantine should be developed. In the event of future outbreaks in which quarantine measures are implemented, a standardized instrument would enable a comparison between the psychological responses to outbreaks of different infectious causes and could be used to monitor symptoms over time.

Despite these limitations, the results of this survey allow for the generation of hypotheses that require further exploration. Our data show that quarantine can result in considerable psychological distress in the forms of PTSD and depressive symptoms. Public health officials, infectious diseases physicians, and psychiatrists and psychologists need to be made aware of this issue. They must work to define the factors that influence the success of quarantine and infection control practices for both disease containment and community recovery and must be prepared to offer additional support to persons who are at increased risk for the adverse psychological and social consequences of quarantine.

## Appendix

### Comments from survey respondents

Unmet informational needs:

**1. Public health /employers:**

a. Difficulty in access: "Called Public Health for 2 days. Got through 3 times; waited on hold for hours, then got hung up on." (respondent # 131)

b. Failed expectations: "I was expecting someone from Public Health to check up on me but never got a call except on my last day of quarantine." (respondent #126); "Nobody told me anything. I was not contacted by health officials at all." (respondent# 99); "My employer should have been more forthcoming." (respondent #7); "I was not called by the hospital I worked at. I saw the quarantine on the news and spent a whole day trying to get through to my unit." (respondent #40)

c. Lack of support: "I was looking for more support from the health care professionals. They left me in the dark to deal with this." (respondent #22)

**2. Nature of information:**

a. Details re: infection control: "I have since learned that there are a lot of precautions that no one ever told me about." (respondent #81)

b. Inconsistencies: "Information was not always the same. Many inconsistencies." (respondent #66)

c. Timing: "Information was given too late, as I started 1 week after exposure. Unacceptable!" (respondent #27)

d. Specific issues:

i. Children: "Nobody can tell me exactly where my children would be arranged to go in case I got SARS myself. I was very panicked at that time and my husband was admitted that time because of the SARS." (respondent # 78)

ii. Onset of symptoms: "What symptoms were considered serious and what to do when I experienced those symptoms." (respondent # 21); "I was mildly alarmed to realize that I didn't know what to do if I actually did develop symptoms of SARS." (respondent # 111)

iii. Prognosis of SARS: "Most of the really important info is largely unknown" (respondent #53); "Prognosis for SARS, how many have recovered, what health problems recovered patients still have." (respondent #8I)

iv. Mode of transmission: "If airborne what were the chances of contracting the disease… MD unable to answer." (respondent #90)

## References

[1] Risse GB. "A long pull, a strong pull and all together": San Francisco and bubonic plague, 1907–1908. Bull Hist Med. 1992;66:260–86. 1596632

[2] Twu SJ, Chen TJ, Chen CJ, Olsen SJ, Lee LT, Fisk T, Control measures for severe acute respiratory syndrome (SARS) in Taiwan. Emerg Infect Dis. 2003;9:718–20. 12781013 10.3201/eid0906.030283 PMC3000163

[3] Centers for Disease Control and Prevention. Update: use of quarantine to prevent transmission of severe acute respiratory syndrome—Taiwan 2003. MMWR Morb Mortal Wkly Rep. 2003;52:680–3. 12881699

[4] Mandavilli A. SARS epidemic unmasks age-old quarantine conundrum. Nat Med. 2003;9:487. 10.1038/nm0503-487 12724741 PMC7095793

[5] Toronto Public Health. Severe acute respiratory syndrome (SARS), 2003 May 29 [cited 2003 Aug 30]. Available from: http://www.toronto.ca/health

[6] Barbera J, Macintyre A, Gostin L, Inglesby T, O'Toole T, DeAtley C, Large-scale quarantine following biological terrorism in the United States: scientific examination, logistic and legal limits, and possible consequences. JAMA. 2001;286:2711–7. 10.1001/jama.286.21.2711 11730447

[7] Markel H. Knocking out the cholera: cholera, class and quarantines in New York City, 1892. Bull Hist Med. 1995;69:420–57. 7549410

[8] Markel H. Cholera, quarantines and immigration restriction: the view from John Hopkins, 1892. Bull Hist Med. 1993;67:691–5. 8312708

[9] Weiss D, Marmar C. The impact of event scale—revised. In: Wilson J, Keane, T, editors. Assessing psychological trauma and PTSD. New York: Guilford; 1997.

[10] Radloff LS. The CES-D scale: a self-report depression scale for research in the general population. Appl Psychol Meas. 1977;1:385–401. 10.1177/014662167700100306

[11] Feinstein A, Owen J, Blair N. A hazardous profession: war, journalists and psychopathology. Am J Psychol. 2002;159:1570–5. 10.1176/appi.ajp.159.9.1570 12202279

[12] Boyd JF, Weissman MM, Thompson WD, Myers JK. Screening for depression in a community sample: understanding the discrepancies between depression symptom and diagnostic scales. Arch Gen Psych. 1982;39:1195–200. 10.1001/archpsyc.1982.04290100059010 7125849

[13] American Psychiatric Task Force for the Handbook of Psychiatric Measures. Handbook of psychiatric measures, 1st ed. In: Yonkers KA, Samson JS. Mood disorder measures. Washington: American Psychiatric Association; 2000. p. 523–6.

[14] Corbin JM, Strauss A. Basics of qualitative research. 2nd ed. Thousand Oaks (CA): Sage Publications;1998.

[15] Kessler RC, Sonnega A, Bromet E. Post-traumatic stress disorder in the National Comorbidity Survey. Arch Gen Psych. 1995;52:1048–60. 10.1001/archpsyc.1995.03950240066012 7492257

[16] Diagnostic and statistical manual of mental disorders, 4th ed. Washington: American Psychiatric Association; 1997.

[17] Breslau N, Kessler RC, Chilcoat HD, Trauma and posttraumatic stress disorder in the community: the 1996 Detroit area survey of trauma. Arch Gen Psych. 1998;55:626–32. 10.1001/archpsyc.55.7.626 9672053

